# Neutrophils in Tissue Trauma of the Skin, Bone, and Lung: Two Sides of the Same Coin

**DOI:** 10.1155/2018/8173983

**Published:** 2018-04-23

**Authors:** A. Kovtun, D. A. C. Messerer, K. Scharffetter-Kochanek, M. Huber-Lang, A. Ignatius

**Affiliations:** ^1^Institute of Orthopedic Research and Biomechanics, Trauma Research Center Ulm, University of Ulm, 89081 Ulm, Germany; ^2^Institute of Clinical and Experimental Trauma Immunology (ITI), Trauma Research Center Ulm, University of Ulm, 89081 Ulm, Germany; ^3^Department of Dermatology and Allergic Diseases, Trauma Research Center Ulm, University of Ulm, 89081 Ulm, Germany

## Abstract

Following severe tissue injury, patients are exposed to various danger- and microbe-associated molecular patterns, which provoke a strong activation of the neutrophil defense system. Neutrophils trigger and modulate the initial posttraumatic inflammatory response and contribute critically to subsequent repair processes. However, severe trauma can affect central neutrophil functions, including circulation half-life, chemokinesis, phagocytosis, cytokine release, and respiratory burst. Alterations in neutrophil biology may contribute to trauma-associated complications, including immune suppression, sepsis, multiorgan dysfunction, and disturbed tissue regeneration. Furthermore, there is evidence that neutrophil actions depend on the quality of the initial stimulus, including trauma localization and severity, the micromilieu in the affected tissue, and the patient's overall inflammatory status. In the present review, we describe the effects of severe trauma on the neutrophil phenotype and dysfunction and the consequences for tissue repair. We particularly concentrate on the role of neutrophils in wound healing, lung injury, and bone fractures, because these are the most frequently affected tissues in severely injured patients.

## 1. Introduction

The severe inflammatory response after major injury is known to contribute critically to primary healing complications or to induce secondary problems in remote organs, which were not affected initially, including in acute respiratory distress syndrome (ARDS), sepsis, and multiorgan failure (MOF). Neutrophils are part of the “first line of cellular defense” and crucially modulate subsequent repair processes after tissue damage. After injury, neutrophils are rapidly recruited to the inflammation site after injury by microbe- and danger-associated molecular patterns (MAMPs and DAMPs, respectively, with MAMPs also known as PAMPs or pathogen-associated molecular patterns). Multiple inflammatory mediators are potent chemoattractants for neutrophils, including C-X-C motif ligand (CXCL) 1–3, macrophage inflammatory protein-1*α*, the anaphylatoxin C5a and leukotriene B_4_ (LTB_4_), and interleukin-8 (IL-8) [1, 2]. Chemoattractants as IL-8 not only promote chemotaxis but also contribute to a mobilization of immature leukocytes by the bone marrow. This release of immature and, therefore, less deformable neutrophils contributes to a subsequent sequestration in distal organs, laying the foundation to harmful side effects of neutrophils [3]. Following severe trauma or during sepsis, antiapoptotic genes are transiently upregulated, increasing the neutrophil circulation half-time [4]. At the injury site, neutrophils themselves produce a significant amount of LTB_4_ [5], phagocytize cellular debris and bacteria, and subsequently may undergo NETosis, forming neutrophil extracellular traps (NETs). Furthermore, they generate reactive oxygen species (ROS), antimicrobial peptides, serine proteases, and various cytokines and chemotaxins, including interleukin- (IL-) 1*β*, IL-6, IL-10, and monocyte chemotactic protein-1 (MCP-1), which, in turn, modulate the inflammatory response and further attract monocytes and macrophages [6] (for a comprehensive review of neutrophil-derived cytokines, see [7]). It is noteworthy that the quantitative contribution of neutrophils to the overall cytokine concentrations may be relatively low in comparison to macrophages. Nevertheless, the neutrophil response contributes to reduced inflammation and ensures adequate tissue repair [8, 9]. The mechanisms of neutrophil-mediated resolution of inflammation include the clearance of DAMPs and the production of anti-inflammatory cytokines, including IL-10 and IL-1Ra [10], and of lipid mediators [11]. In addition, neutrophils degrade inflammatory cytokines by aggregated NETs, secrete soluble factors, including azurocidin, cathepsin G, lipoxins, and lysophosphatidylserine, and are able to reprogramme macrophages to the regulatory M2 phenotype [6, 12–15].

However, in the case of excessive posttraumatic inflammation, neutrophils may become overactivated or dysfunctional. Consequently, they secrete an altered cytokine profile, increase ROS production, and undergo massive NETosis, thereby aggravating tissue damage and even harming surrounding healthy tissues [15–18]. The majority of studies evaluating neutrophil dysfunctions after trauma address their impaired antimicrobial defense and role in sepsis development [19, 20]. This review focuses on the roles of neutrophils in those organs that are frequently initially affected in traumatized patients: skin, lungs, and bones.

## 2. Trauma-Induced Phenotype Changes and Functional Consequences

Trauma and subsequent complications affect the phenotype and function of circulating neutrophils, and, particularly, in case of severe trauma, the development of dysfunctional neutrophils might play a detrimental role [21, 22]. Indeed, severe posttraumatic inflammation induces a boost in the release of banded and immature neutrophils into the circulation, leading to bone marrow exhaustion and a compromised immune response, both associated with a poor outcome [21, 23, 24]. Additionally, morphological changes were observed after trauma, including increased cell size and membrane plasticity and a modified shape, wherein neutrophils become more elongated [25, 26]. Within the population of neutrophils, there is a degree of heterogeneity that has received growing attention since the 1980s (see [27] for a summary of currently described neutrophil subsets). Until today, there is no certainty to what extent neutrophil heterogeneity is biologically relevant [27, 28]. However, as trauma induces not only an activation of neutrophils, partly accompanied by an extended life span of certain subsets, but also a rapid recruitment of naïve cells as well as an emergency granulopoiesis, trauma itself might contribute to neutrophil heterogeneity [29]. For example, in trauma, there are immunosuppressive low-density neutrophils (LDNs), a subtype of neutrophils named after their discovery in the fraction of the peripheral blood mononuclear cells (PMBC) [29, 30]. These granulocytes are not only activated but express a high level of arginase activity, which in turn might be linked to T-cell function providing an interesting modulation and possible impairment of the adaptive immunity mediated by neutrophils during trauma [30]. In sepsis, it has been demonstrated that this granulocyte subset inhibits T-cells, possibly via arginase release and/or ROS production [29, 31, 32]. In contrast, there might be subsets of neutrophils, which are beneficial to repair the initial trauma impact. For example, a population of CD11b^+^/Gr-1^+^/CXCR4^hi^ neutrophils likely recruited by VEGF-A induce revascularization via MMP-9 [33]. While neutrophil heterogeneity is often described in the context of chronic inflammation, for example, caused by cancer [27, 29], research in the trauma context to elucidate the diametral effects of the neutrophil collectively represents a promising field, which, however, is beyond the scope of this review.

The egress of neutrophils from the bone marrow and their recruitment to the injured tissue is crucial for mounting an adequate inflammatory response. The impairment of targeted chemotaxis has been described in many inflammatory disorders, including diabetes mellitus and viral infections (e.g., HIV and influenza) [34–36]. Adequate chemokinesis is ensured by sufficient expression of surface receptors, including the IL-8 receptors CXCR1 and CXCR2, Fc*γ*RIII (CD16), IL-6 receptor (IL-6R), and complement receptor C5aR1 [37]. Indeed, trauma is associated with reduced expression of CXCR1, CXCR2, and C5aR1, all of which may be partially internalized by or released from neutrophils in microvesicles [38–40]. IL-6R is actively shed from the neutrophil surface to induce IL-6 transsignaling, which amplifies the inflammatory effects of IL-6 [41], and to regulate T-cell responses [42, 43]. Overall, these trauma-induced functional changes may desensitize neutrophils towards persisting danger.

Killing of phagocyted pathogens in neutrophils is ensured via two distinct mechanisms. One is oxygen based and executed via the formation of ROS, whereas the other is oxygen independent [37]. In trauma, neutrophils produce increased amounts of ROS and increase the expression of gp91^PHOX^, a membrane-residing subunit of nicotinamide adenine dinucleotide phosphate (NADPH) oxidase, a key enzyme in ROS production [44, 45]. The enhanced ROS response might contribute to the damage of the endothelial barrier and induce vascular leakage, resulting in further complications, including edema and organ dysfunction, for example, ARDS [44, 46]. Oxygen-independent mechanisms include the release of neutrophil granules containing digestive serine proteases, for example, neutrophil elastase, cathepsin G, proteinase 3, and azurocidin [47, 48]. The release of proteases is regulated by the intraphagosomal pH, which, upon improper activation after injury, may lead to impaired protease activation and disturbed microbial killing [49]. Proteases released by neutrophils likely act predominantly locally, as the clearance capacity of antiproteases such as *α*_2_-macroglobulin is sufficient to degrade the listed enzymes in a systemic dimension and is increased in scenarios of severe inflammation [50, 51].

**Figure 1:**
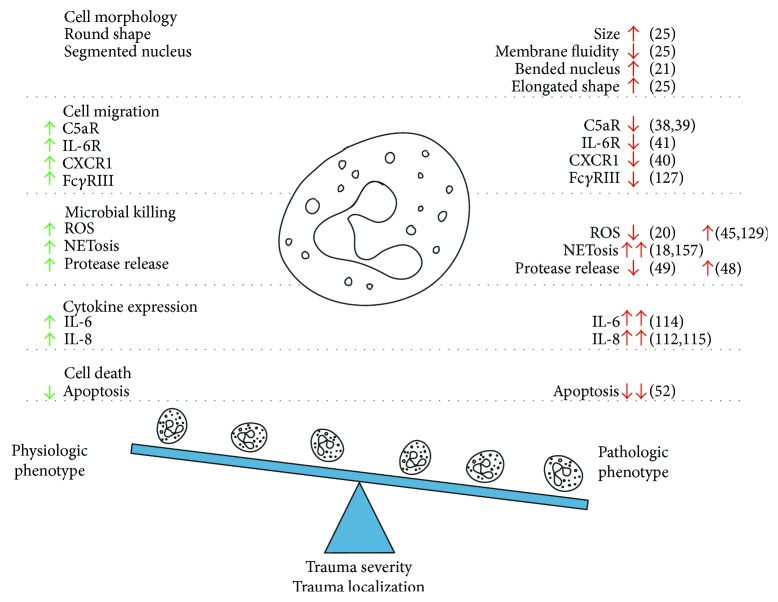
Trauma-induced changes in neutrophil phenotype lead to neutrophil overactivation and dysfunction, thus negatively affecting migration and maturation, impairing antimicrobial defense and clearance of cell debris, and delaying resolution of inflammation.

Apoptosis and NETosis represent mechanisms of programmed death of neutrophils. Inflammatory stimuli may prolong the circulation half-life of neutrophils from 6 h up to several days based on the upregulation of antiapoptotic proteins, including induced myeloid leukemia cell differentiation protein Mcl-1, and a reduced level of proapoptotic proteins, including apoptosis regulator Bax [11, 52]. However, the functional capacity of such neutrophils remains questionable. NETosis is a mechanism of extracellular neutrophil-mediated killing after cell death. NETs consist of fibrils containing ROS, DNA, chromatin, and granular proteins and are released by active expulsion via an NADPH oxidase-dependent mechanism. Although NETosis is believed to induce programmed cell death, recent data imply that neutrophils may remain viable afterwards [53]. Because NET-mediated destruction is unspecific, excessive NETosis is thought to contribute to tissue damage after trauma [54, 55]. Trauma-induced changes in neutrophil phenotype and functions are summarized in Figure 1.

## 3. Neutrophil Actions in Specific Trauma Settings

Neutrophil functions may depend on the micromilieu of the damaged tissue. Confirming this, different trauma models frequently produced contradictory results regarding neutrophil functions in different organs. For example, in a model of severe injury, neutrophil depletion did not improve bone regeneration [56], but did mitigate pulmonary damage [17, 57]. Interestingly, a recent study showed that fracture-associated mitochondrial DAMPs may “prime” pulmonary neutrophils, thereby desensitizing them towards pathogens and impairing the pulmonary response to lung infection [58]. These findings could be explained by the compartmentalization of the immune response and by different expression patterns of inflammatory mediators and adhesion molecules in various tissues. Indeed, as already reviewed elsewhere [59], distinct tissues and cell types contribute differently to the production of inflammatory mediators in trauma and sepsis. For example, in sepsis, tumor necrosis factor *α* (TNF*α*) is predominantly expressed in the liver, spleen, and lungs by Kupffer cells, leukocytes, and lung epithelial and immune cells, respectively. Additionally, in downstream signaling, for example, in nuclear factor kappa-light-chain-enhancer of activated B cell (NF-*κ*B) activation, the highest activities were observed in the skin, lungs, and spleen, with minor involvement of the liver, kidney, and heart [60]. Because many inflammatory mediators are important chemotaxins for neutrophil recruitment, it is unsurprising that different organ injuries result in different local and systemic inflammatory patterns. Another possible explanation might be the organ-specific expression of different adhesion molecules, including intercellular adhesion molecule 1 (ICAM-1), vascular cell adhesion protein 1 (VCAM-1), selectins, and CD11b, which are important for the neutrophil influx from the blood vessels into the tissue by mediating their adhesion, rolling, and subsequent migration [59].

In this review, we concentrate on the most frequently injured organs: the skin, as a first target for surface damage; the lungs, which represent a frequent target and major effector organ in trauma, because they are also actively involved in hematopoiesis and coagulation [61]; and the bone, which has a unique micromilieu due to the enclosed bone marrow.

### 3.1. Role of Neutrophils in Wound Healing

The skin is the first body barrier and is the most frequently injured in trauma. Because skin wounds allow pathogen access to the body, they require an efficient clearing of pathogens and a rapid healing process. Wound healing consists of the interconnected phases of hemostasis and inflammation, tissue regeneration, and remodeling. Hemostasis is initiated within minutes after injury and is accompanied by inflammation and platelet activation, resulting in a stable fibrin clot with an active neutrophil influx [8, 9, 62, 63]. In wounds, neutrophils are recruited by proinflammatory cytokines, including TNF*α*, growth factors, including platelet-derived growth factor (PDGF) and transforming growth factor *β* (TGF-*β*), and arachidonic-acid derivates, including leukotrienes and prostaglandins. Furthermore, neutrophils are attracted by the complement anaphylatoxins C3a and C5a [8, 48, 64, 65]. The physiological role of neutrophils in wound healing does comprise the clearance of not only pathogens but also the abundant erythrocytes [66]. The role of neutrophils in the downstream repair processes remains unclear. On the one hand, neutrophils do not enhance collagen synthesis or granulation tissue formation [67]. Wound healing in germ-free mice, fetuses, and oral mucosa is associated with lower neutrophil-driven inflammation and scarless regeneration, which demonstrates the benefits of a limited neutrophil involvement [64, 68–70]. Additionally, the reduced presence of neutrophils in germ-free lesions correlated with increased levels of the anti-inflammatory cytokine IL-10 and vascular endothelial growth factor (VEGF) and was associated with an accelerated wound epithelialization [68]. On the other hand, in wounds, neutrophils express cytokines, among others TNF*α*, which can contribute to reepithelialization and wound closure [71, 72]. Furthermore, stimulated neutrophils secrete VEGF, which may contribute to wound healing by encouraging angiogenesis [73]. The process of efficient wound healing also requires neutrophil clearance [48, 74], and it was shown that macrophage stimulation promoted neutrophil removal and wound healing [75]. Indeed, after clearance of MAMPs and DAMPs, neutrophils—via *β*2 integrins [76]—are phagocyted by macrophages and this is a very strong signal for the macrophage to release TGF-*β*1. TGF-*β*1 stimulates differentiation of myofibroblasts, which contribute not only to wound contraction but also to a collagen synthesis [77].

While the presence of neutrophils is generally restricted to the inflammatory phase, it can be prolonged by physical trauma and/or ongoing contamination, thus exerting deleterious effects and inhibiting efficient wound healing [62, 74, 78]. DAMPs and MAMPs combined with cytokine release after trauma further extend the inflammatory response of neutrophil in wounds, among others via NF-*κ*B signaling [79, 80]. The toxic arsenal of neutrophils primarily directed against pathogens leads to collateral damage via distinct mechanisms—particularly, when released as a consequence of necrosis rather than apoptosis. These unwanted side effects damage the extracellular matrix and affect clotting and further mechanisms that are involved in wound healing [48, 62, 81]. The harmful potential of neutrophils is further reflected in the setting of second hits, including in reperfusion injury, which has been demonstrated to increase the invasion of neutrophils, thereby leading to sustained inflammation [82]. Another example of unsolicited effects of neutrophils is excessive NETosis, which has been described as an inhibitor of wound healing in diabetes patients [18]. There are several mechanisms to control neutrophil effects and induce repair. For example, radicals generated by hyperactivated neutrophils are cleared via superoxide dismutase 3 (SOD3) from mesenchymal stem cells (MSCs) [83]. In addition, mesenchymal stem cells can decelerate neutrophil migration via IL-10 and TNF-stimulated gene/protein-6 [84]. Furthermore, epidermal growth factor as part of the saliva lessens neutrophil recruitment and activity, explaining a beneficial effect of wound licking in animals [85].

By contrast, neutrophils also have many positive effects in wound healing. For example, neutrophils counterbalance hyperproliferation, thereby preventing malignancy [64]. From an evolutionary point, the wound-healing mechanism developed when wounds were more likely to be contaminated. Therefore, a pronounced inflammatory response with neutrophils at the wound site neutralizing bacterial intruders might have been crucial to allow for subsequent keratinocyte proliferation [64]. Moreover, neutrophils are required to keep the commensal microbiota in check [68]. Furthermore, delayed healing of infected wounds supplies proliferating skin cells with sufficient oxygen. The oxygen also acts as bactericide and is a prerequisite for neutrophil ROS generation [86]. Additionally, neutrophils support an additional recruitment of macrophages and T-cells by upregulation of MCP-1 and chemokine ligand 3 (CCL3) [4]. The release of carbonic anhydrases by neutrophils alters the wound microenvironment, which supports healing processes under compromised conditions [87].

In summary, neutrophils contribute to the clearing of DAMPs and MAMPs in nonsterile skin lesions, thereby promoting wound healing. However, the presence and activity of neutrophils require tight regulation, which is a challenge, particularly in the setting of severe trauma.

### 3.2. Role of Neutrophils in Lung Injury

The lung is a unique organ with respect to neutrophil migration, resulting in high neutrophil numbers even in healthy humans. There is growing evidence that under physiological conditions, peripheral-activated neutrophils are cleared and deprimed in a healthy lung [88, 89]. In contrast to other tissues, neutrophils do migrate not only in high endothelial venules via *β*_2_-integrin but also in the alveolar capillary bed via a L-selectin- and *β*_2_-integrin-independent pathway [90–94]. The capillaries' interwoven network results in a high concentration of neutrophils in the pulmonary vessels compared to blood in the large vessels, which might explain partially the vulnerability of the lung against neutrophil-mediated tissue injury [88, 90, 91, 95]. Another hypothesis emphasizes the role of the lung as a control site for primed neutrophils. If overloaded, the lung might lose its property as site of surveillance and depriming but might even contribute to it [89]. The small diameter of capillary segments (approximately 5 *μ*m) compared with the size of a neutrophil (approximately 7-8 *μ*m), on the one hand, improves neutrophil contact with the vascular wall, thereby facilitating extravasation, but, on the other hand, requires a high degree of cellular deformability [90, 96]. Neutrophil deformability is modulated by chemotactic factors, including anaphylatoxin C5a [25, 97] and chemotactic tripeptide fMLF (N-formylmethionyl-leucyl-phenylalanine, previously known as fMLP) [98–100], and by various bacterial compounds, including lipopolysaccharides (LPS) [25, 101]. Transient pulmonary overfishing of neutrophils results in sequestration within the lungs and might contribute to a succeeding reduced cell count in the blood, particularly during the early stage of pulmonary inflammation [97, 101]. Another characteristic of the capillary bed of the lungs are tricellular corners. There, three endothelial cells intersect, building discontinuous tight junctions. Therefore, they provide a possibility to migrate around instead of through endothelial tight junctions, thus contributing to >75% of neutrophil extravasation when stimulated, for example, with IL-1 [102]. In healthy humans, the stimulation of neutrophil pulmonary extravasation by LTB_4_ without further significant inflammatory impact does not cause deterioration in pulmonary barrier permeability, which indicates that physiologically, neutrophils can extravasate without harming the barrier [103]. Accordingly, neutrophils do not require matrix metalloproteinase or serine protease for pulmonary extravasation [104]. In conclusion, in the lungs, neutrophils display unique migration mechanisms, resulting in a large neutrophil number, which is highly relevant in trauma.

ARDS (with mild ARDS being a term for acute lung injury (ALI)) is defined as an “acute diffuse, inflammatory lung injury” caused by primary pulmonary factors (e.g., pneumonia and pulmonary contusion) or secondary harmful events (e.g., polytrauma, shock, burns, and aspiration) [105, 106]. Among trauma patients, mild and severe ARDS occur in 4% and 12%, respectively, and are associated with a longer intensive care unit stay and increased hospital costs [107]. A characteristic of ARDS is severe hypoxemia, which is caused by the leakage of pulmonary vessels with the recruitment of neutrophils, a marked right-to-left shunt and an increased dead space as well as a decrease of pulmonary compliance and a dysfunctional pulmonary epithelium [106]. Although there is numerous data on ARDS and neutrophils [90, 93, 94], the exact role of these cells in ARDS remains poorly understood. In ARDS, inflammatory mediators, including IL-1*β*, IL-6, and IL-8, which are abundantly secreted by type-2 alveolar cells, macrophages, and endothelial cells after blunt chest trauma, induce a hyperactivation of neutrophils [17, 93, 94, 108, 109]. High levels of IL-6 and IL-8 are risk factors for ARDS development after trauma [110, 111]. In traumatic injury, neutrophil activity in general is associated with elevated levels of IL-6, IL-8, and TNF*α*, but also of IL-10, and, simultaneously, a reduced antimicrobial defense [112–115]. The pulmonary inflammatory mediators further enhance neutrophil activity and their deleterious effect on the endothelium and epithelium. Thereby, they increase transcellular permeability, contributing to lung edema and poor ARDS prognosis [17, 92]. Whereas endothelial cell damage is ROS dependent, epithelial cells might be more resistant towards radicals, but like endothelial cells, they are also affected by activated, adhering neutrophils [116].

Several studies used a neutrophil depletion approach to define the role of neutrophils in trauma. Neutrophil depletion in trauma-induced ARDS was associated with higher chemokine levels in the bronchoalveolar lavage fluid, including granulocyte colony-stimulating factor (G-CSF), and led to an improved outcome [17, 117]. In addition, neutrophil deficiency resulted in reduced IL-1*β*, MIP-2, and TNF*α* levels in a mouse hemorrhagic shock model, which underlines the role of neutrophils contributing to pulmonary inflammation [118]. In the absence of neutrophils, some protective effects of the lung-blood barrier were described [17, 119]. Further harmful effects of neutrophils include proteolysis of endo- and epithelial cadherins and attacking the endothelial barrier [120, 121]. In a murine influenza aspiration-induced ARDS model, blockade of neutrophil recruitment via inhibition of the CXCL10-CXCR3 axis resulted in an improved outcome and survival [122]. Furthermore, patients recovering from neutropenia are at risk for ARDS because “reappearing” neutrophils provoke inflammation [123].

However, there are several studies, mainly on infectious- and less in trauma-induced shock, demonstrating that neutrophils are not the only “scapegoat”, as pulmonary trauma activates other components of the innate immunity, for example, alveolar macrophages, as well as the coagulation system [124]. For example, neutrophil elastase inhibition did not reduce mortality after ARDS [125]. Another study comparing endotoxin- and bacteria-induced ARDS rat models found that bacteria-triggered ARDS was associated with a poorer outcome, although alveolar neutrophil influx and activity (as determined by elastase or ROS production) were similar. This indicates that there are further factors in addition to neutrophil actions in ARDS development [126]. Furthermore, there is evidence that blunt chest trauma without a second hit induces a transient short-term neutrophil activation with a significant reduction of CXCR2 and C5aR and a mobilization of young (Fc*γ*RIII-low) neutrophils [127, 128]. Lacking a strong second inflammatory stimulus, for example, subsequent sepsis or pneumonia, inflammation regresses without causing ARDS or MOF, implying a vulnerable phase after trauma-induced immune activation [127, 129, 130].

### 3.3. Role of Neutrophils in Bone Fracture Healing

Approximately 30% of severely injured patients (injury severity score (ISS) > 16) have concomitant fractures of the extremities [131]. These patients are at a high risk of delayed bone healing or nonunion formation, because of systemic hyperinflammation associated with severe trauma [132–134]. Fractures heal by three partially overlapping phases: the initial inflammatory phase, the repair phase comprising soft callus formation and intramembranous and endochondral ossification, and the remodeling phase, where the initially woven bone is converted to a lamellar bone until the original bone shape is restored [135]. The initial local inflammation starts with rapid hematoma formation, which serves as a scaffold for immune and progenitor cells, initiating regeneration [135]. Neutrophils are the most abundant cells in the early fracture hematoma [136]. Initially, they originate from the blood, leaking from the ruptured vessels. Then they actively migrate from the bloodstream into the damaged bone within minutes after fracture. Moreover, neutrophils or their progenitors can invade the hematoma directly from the damaged bone marrow. Indeed, Hoff et al. reported that, immediately after injury, the fracture hematoma mainly contains bone marrow cells, the majority being CD16^+^-immature granulocytes [136]. Within 72 h, either maturation of these granulocytes or invasion of CD16^+^-mature granulocytes from the circulation occurs [136]. Notably, the bone marrow at the fracture site becomes actively involved, because CD16^+^ cells are increasingly found there, indicating general bone-marrow activation in response to injury. The neutrophil numbers rapidly increase at the fracture site during the early inflammatory phase and then slowly subside until day 7–10, when only a few cells are observed in the soft periosteal callus [56, 137, 138].

In uneventful bone healing after isolated fracture, there is a continuing debate over the role of neutrophils [56, 132]. Some authors postulated a negative influence of neutrophils on bone regeneration, because their depletion from the bloodstream improved fracture healing, as confirmed by radiological examination and improved mechanical properties of the healed femur [139]. It was proposed that neutrophils would induce tissue damage by secreting collagenase, elastase, free radicals, and arachidonic acid and that the neutrophil-induced inflammatory response would aggravate the already existing ischemia, leading to edema and a local circulatory shutdown [139]. Others found that neutrophil depletion promoted osteogenic but suppressed chondrogenic differentiation of progenitor cells in a model of growth plate injury; however, the mechanisms were not elucidated [138]. This might be beneficial for intramembranous bone formation, but implies that diaphyseal fracture healing might be delayed, because in this case, cartilaginous callus formation is essential. Interestingly, the authors did not observe any significant influence of neutrophil depletion on the early immune response after fracture, because monocyte and lymphocyte infiltration and IL-1*β* and TNF*α* expression at the injury site were unaffected [138]. Fracture healing was also impaired after zymosan-stimulated ROS production in a rat fracture model [140].

By contrast, stimulation of neutrophil recruitment by G-CSF supported fracture healing. The biomechanical properties of the healed bones were improved [141, 142], bone formation was increased [143], and the expression of angiogenic (angiopoietin, VEGF) and osteogenic (bone morphogenetic proteins- (BMP-) 2 and BMP-4) factors in the fracture callus was enhanced by G-CSF treatment [142]. However, G-CSF does not only promote neutrophil egress into the bloodstream but also facilitate bone marrow stem cell and preosteoblast recruitment to the injury site. Furthermore, it enhances VEGF release and the recruitment of CD34^+^ cells, which contribute to angio- and vasculogenesis [143]. This may improve neovascularization and bone formation independently of enhanced neutrophil recruitment [142, 143].

More recent studies demonstrated that a balanced neutrophil activation may be important for undisturbed fracture healing. After neutrophil depletion with Ly-6G antibody, the recruitment of monocytes and macrophages to the fracture site was disturbed and the concentration of inflammatory mediators, including IL-6, IL-10, CXCL1, and MCP-1, in the fracture hematoma was altered [56, 144]. Subsequent bone regeneration was considerably disturbed in neutrophil-depleted mice. These findings imply that neutrophils crucially regulate the immune response at the fracture site, resolve inflammation, and induce downstream responses, which are essential for successful bone repair. Supporting this, Bastian et al. proposed that neutrophils may form “emergency extracellular matrix” consisting of fibronectin in the initial fracture hematoma, which could serve as a scaffold for stromal cell recruitment, thereby promoting healing [137]. The authors reported that early neutrophil recruitment to the fracture hematoma was associated with fibronectin synthesis. Moreover, neutrophils could be positively costained for fibronectin. Interestingly, the overall cell number in the fracture hematoma was unchanged from days 3 to 10, whereas subpopulation analysis showed that neutrophil numbers diminished, implying that other cell populations, presumably macrophages and stromal cells, invade the fibronectin matrix. At the same time, the fibronectin content was unchanged, whereas the collagen type-1 content increased, indicating that collagen is produced by these newly recruited cells [137]. Therefore, these recent findings support the hypothesis that neutrophils are essential for undisturbed bone regeneration, at least in uneventful bone fracture.

Whether neutrophils play a role in compromised fracture healing associated with severe trauma remains unclear. Several studies found enhanced neutrophil and diminished macrophage recruitment to the fracture hematoma in a rodent model of severe injury, implying that neutrophils might be involved in the pathogenesis of impaired bone healing after trauma [56, 145, 146]. By contrast, bone healing was not improved in a mouse model of combined fracture and thoracic trauma when neutrophils were depleted, suggesting that they may play only a minor role or were dysfunctional in this scenario [56]. The latter suggestion could be confirmed by a recent clinical study of Bastian et al., who reported altered leukocyte kinetics in severely injured patients with subsequent fracture-healing complications [22]. These patients exhibited impaired systemic neutrophil and monocyte mobilization, indicating immune exhaustion.

Even if the current literature is very limited and in part greatly debated, it is clear that neutrophils play a major role in the initial immune response after fracture and initiate downstream responses leading to bone repair. However, further research is necessary to elucidate their role in bone regeneration and the pathogenesis of fracture-healing complications associated with severe trauma.

## 4. Neutrophils as a Therapeutic Target in Trauma

To utilize the potent defensive mechanisms and clearance capacity for MAMPs and DAMPs by neutrophils in the initial posttraumatic response, enhanced recruitment of neutrophils via G-CSF-based therapeutics, including filgrastim, has been postulated as a rational therapy [147]. Indeed, in the clinical setting of tissue damage after major surgery, G-CSF-treatment provoked reinforcement of the systemic innate immune response and reduced septic complications [148]. After acute traumatic brain injury, G-CSF application reduced bacteremia, although overall survival was not improved [149]. However, contradictory effects were reported concerning local healing: In a rodent model of full-thickness supraspinatus tendon defects, G-CSF treatment locally increased cellularity after rotator cuff repair, but failed to improve structural healing [150]. By contrast, accelerated wound healing was found after topical G-CSF application [151]. In a mouse model, the transcriptional coregulator B cell leukemia/lymphoma 3 (Bcl3) was identified to downregulate emergency granulopoiesis as consequence of a transplant-mediated ischemia/reperfusion lung injury, limiting pulmonary damage [152]. In another approach to mitigate neutrophil recruitment, a porcine burn wound model proposed reduced neutrophil activity by the application of atorvastatin [153]. Likewise, attenuation of neutrophil recruitment by neutralization of IL-8 alleviated neutrophil invasion and damage to the lung [154]. Certainly, more research is necessary to define the exact indications after tissue trauma and the dosing, timing, and application route of such approaches.

By contrast, inhibition of extensive neutrophil activation has also been proposed to prevent the collateral damage by neutrophils. For example, in a murine blunt chest injury model with lung contusion, neutrophils and their oxidative response have been identified as a major contributor to acute lung injury and neutrophil depletion was protective [155]. Another experimental study demonstrated the beneficial effect of valproic acid, which reduced neutrophil influx and reduced tissue damage via decreased MPO activity, however with partial immunosuppression [156]. In a mouse model of LPS-induced ARDS, systemic application of mesenchymal stem cells reduced neutrophil recruitment and activity (e.g., NETosis), improving overall survival [157]. Whether the MSCs as cells or parts of their secretome induced these effects remains to be investigated. Leukocyte filtration strategies were also examined in numerous clinical studies, particularly in the context of major cardiac surgery. There is evidence that pulmonary, cerebral, and renal function may improve by neutralization of activated neutrophils using filtration [158, 159]. However, global neutrophil inhibition after severe tissue trauma is certainly irrational and unsafe, because these cells are major contributors of the “first line of defense” to clear the MAMP and DAMP load. Further research needs to determine which specific markers may indicate host-damaging-activated neutrophils. It is also of interest as to which removal strategies should be followed to beneficially modulate the neutrophil immune response after trauma and to induce an effective regenerative process. Future strategies should also account for the different microenvironmental changes after trauma and the compartmentalization of the neutrophil immune response [59]. Therefore, it might be of importance to either enhance or suppress the local neutrophil response, for example, in the fracture hematoma during fracture healing or in the alveolar space after lung contusion. Therefore, organ compartment-targeted neutrophil therapy may represent a promising future scientific and clinical field.
